# A Review and Benchmark Study of Multi-Scale Entropy Methods for the Fault Diagnosis of Rotating Machinery

**DOI:** 10.3390/e28080851

**Published:** 2026-07-30

**Authors:** Xianzhi Wang, Yang Zhang, Yu Wei, Chenyang Ma

**Affiliations:** 1 School of Automation, Xi’an University of Posts and Telecommunications, Xi’an 710121, China; cagallilan@xupt.edu.cn (X.W.); zhangyang06@stu.xupt.edu.cn (Y.Z.); weiyu@xupt.edu.cn (Y.W.); 2 School of Computer Science and Technology, Xi’an University of Posts and Telecommunications, Xi’an 710121, China

**Keywords:** benchmark study, fault diagnosis, multi-scale entropy, rotating machinery

## Abstract

Multi-scale entropy is a powerful analytical tool that extends single-scale entropy analysis into a multi-scale feature extraction approach, enabling more reliable fault diagnosis in complex machinery by revealing hidden dynamic information that single-scale metrics neglect. Driven by advances in symbolic dynamics, statistical processing, and robustness enhancement, multi-scale methods have evolved into more than 80 variants. Despite this diversity, a comprehensive and systematic evaluation of these methods is still lacking, particularly with regard to the trade-off between diagnostic accuracy and computational efficiency. This study provides a comprehensive benchmark of 85 multi-scale methods evaluated on the XJTU-Gearbox dataset to establish an evidence-based roadmap that balances diagnostic accuracy and computational cost. Through performance comparison and algorithmic-principle analysis, this study elucidates the mechanisms underlying the performance differences among these variants, highlighting how advanced multi-scale methods preserve richer fault information and enhance feature separability. Furthermore, this work identifies promising development trajectories and offers a basis for future integration of multi-scale methods with deep learning models. To avoid overgeneralization, the results are interpreted as a controlled single-dataset benchmark rather than a universal ranking across all datasets, classifiers, operating conditions, or fault severities.

## 1. Introduction

Entropy-based metrics serve as effective tools for monitoring machinery health by quantifying the complexity of dynamic signals [1,2,3,4,5]. Under normal operating conditions, vibration responses are typically dominated by background noise and small disturbances, whereas fault-related states often introduce more structured periodic or harmonic components associated with specific mechanical defects. The resulting differences in signal regularity can be reflected in entropy values and used as diagnostic features. However, single-scale analysis is often insufficient for complex systems because diagnostically useful information is frequently distributed across multiple temporal scales [6,7].

**Figure 1:**
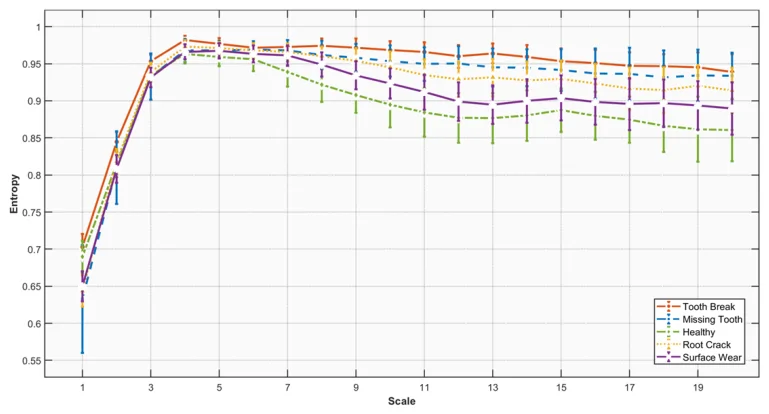
MDE error-bar curves for five gearbox conditions in the XJTU-Gearbox dataset.

Figure 1 illustrates this limitation using the XJTU-Gearbox dataset. The multi-scale diversity entropy curves for the healthy state and four fault types overlap substantially at scale 1, indicating weak separability under single-scale analysis. As the scale factor increases, the trajectories diverge and class separation improves markedly, especially beyond scale 5. Similar improvements in class separability and diagnostic robustness have also been reported in recent fault diagnosis studies [8,9,10,11,12,13,14]. This behavior indicates that multi-scale analysis can expose fault information that remains obscured at a single resolution.

**Figure 2:**
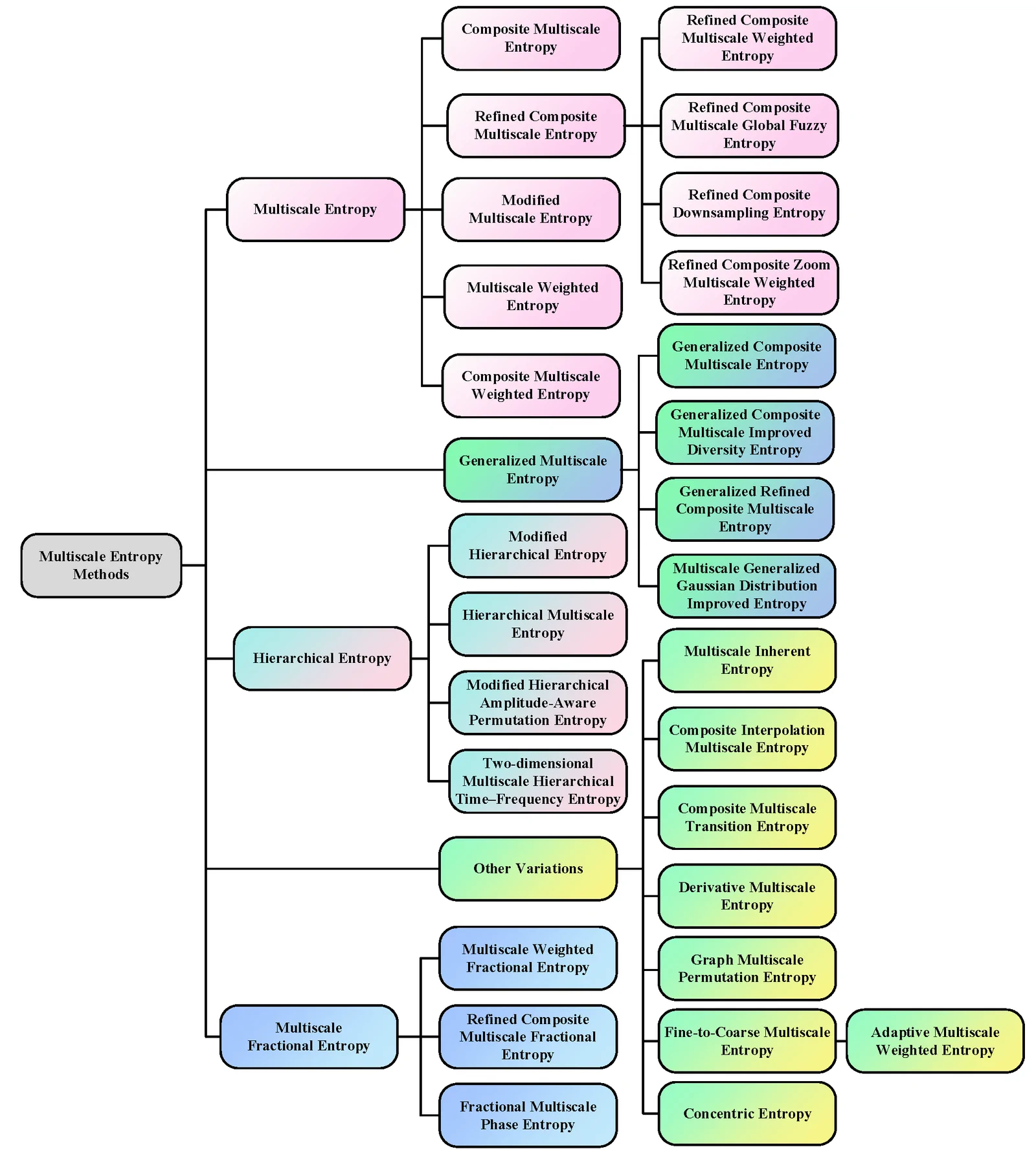
Taxonomy of multi-scale methods.

The success of multi-scale analysis has produced a large family of methods, including composite, generalized, hierarchical, and fractional entropy variants, as summarized in Figure 2 [15,16,17,18]. Although this diversity broadens the analytical toolbox, it also complicates method selection. In particular, the trade-off between diagnostic accuracy and computational cost has not been systematically benchmarked, and the algorithmic sources of performance differences remain unclear.

This study addresses that gap through a unified benchmark of multi-scale entropy methods for rotating machinery fault diagnosis. The benchmark compares diagnostic accuracy and computational cost, identifies the design mechanisms that most strongly influence performance, and distills practical guidance on both effective and inefficient methodological directions.

Compared with existing review papers and isolated comparative studies, the present work is intended as a controlled benchmark rather than a new entropy algorithm. Its main contribution is to evaluate a broad set of multi-scale entropy implementations under the same dataset, feature vector construction, classifier, train/test split, repeated-run protocol, and runtime-recording procedure. Therefore, the benchmark provides more than an accuracy list: it jointly compares diagnostic accuracy, stability, feature extraction time, parameter settings, and practical selection criteria for rotating machinery fault diagnosis.

The scope of the conclusions is also clarified here. Because all experiments are conducted on the XJTU-Gearbox dataset, the ranking of individual methods should be interpreted as evidence under this controlled setting. Cross-dataset generalization to CWRU, Paderborn, MFPT, or other public datasets remains an important future validation step.

The remainder of the paper is organized as follows. Section 2 reviews the investigated methods. Section 3 describes the dataset and evaluation protocol. Section 4 presents the benchmark results and interprets the main design mechanisms. Section 5 discusses future directions, and Section 6 concludes the paper.

## 2. Multi-Scale Methods and Variants

This section reviews the multi-scale entropy families included in the benchmark. Because the study is benchmark-oriented rather than derivation-oriented, the main text retains one canonical formulation per family and describes secondary variants through operator-level differences. When the source literature introduced only a PE-, FE-, or DE-based realization, the benchmark applied the same transferable front-end operator structure to other compatible back-ends for controlled comparison; such benchmark instantiations are explicitly noted in the relevant subsections.

### 2.1. Canonical Multi-Scale Methods

This family starts from the standard mean-based coarse-graining scheme and then modifies either the path generation rule, the entropy statistic, or the way multi-path information is aggregated.

#### 2.1.1. Baseline Multi-Scale Entropy

Multi-scale entropy (MSE) measures complexity across temporal scales by coarse-graining the original series [4,19]. For scale factor τ, consecutive non-overlapping windows of length τ are averaged to form the coarse-grained sequence, whose j-th element is defined as:

(1) $$y_{j}^{\left(\tau \right)}=\frac{1}{\tau }\sum_{i=\left(j-1\right)\tau +1}^{j\tau }x_{i}$$

**Figure 3:**
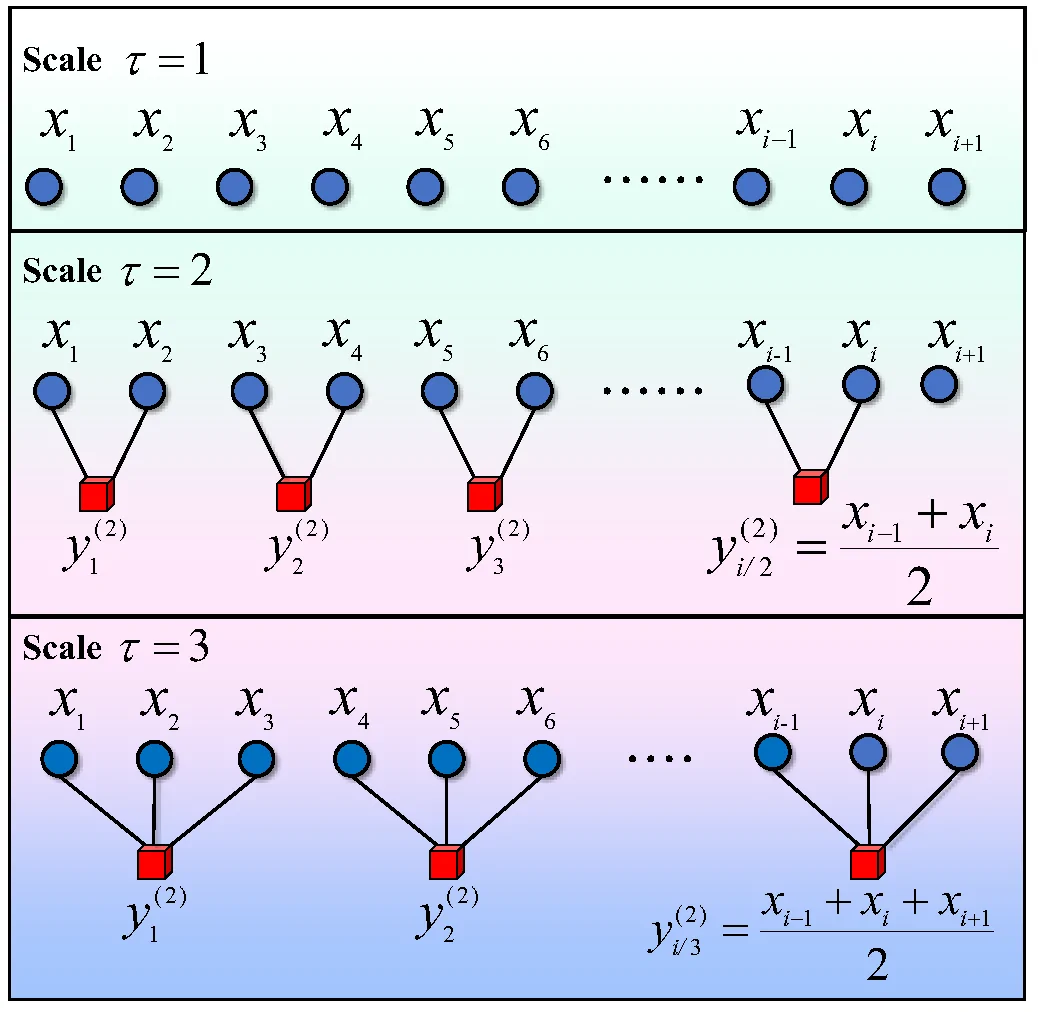
Standard coarse-graining at scale τ.

The resulting sequence has length N/τ. When τ = 1, the coarse-grained sequence reduces to the original time series. The standard coarse-graining procedure is illustrated in Figure 3.

A single-scale entropy measure is then computed on each coarse-grained sequence [20]:

(2) $${MSE}_{t}\left(X,m,p,\tau \right)=E_{t}\left(y^{\left(\tau \right)},m,p\right)$$

Here, t∈{DE, FE, PE}, m is the embedding dimension, and p denotes entropy-specific parameters. DE, FE, and PE differ only in whether the underlying statistic is a diversity-pattern distribution, a fuzzy similarity function, or an ordinal pattern distribution [21,22,23].

#### 2.1.2. Modified Multi-Scale Entropy

Modified multi-scale entropy (MME) addresses data shortening by replacing non-overlapping averaging with a moving-average window [24].

(3) $$y_{\mathrm{mod},j}^{\left(\tau \right)}=\frac{1}{\tau }\sum_{i=j}^{j+\tau -1}x_{i}$$

**Figure 4:**
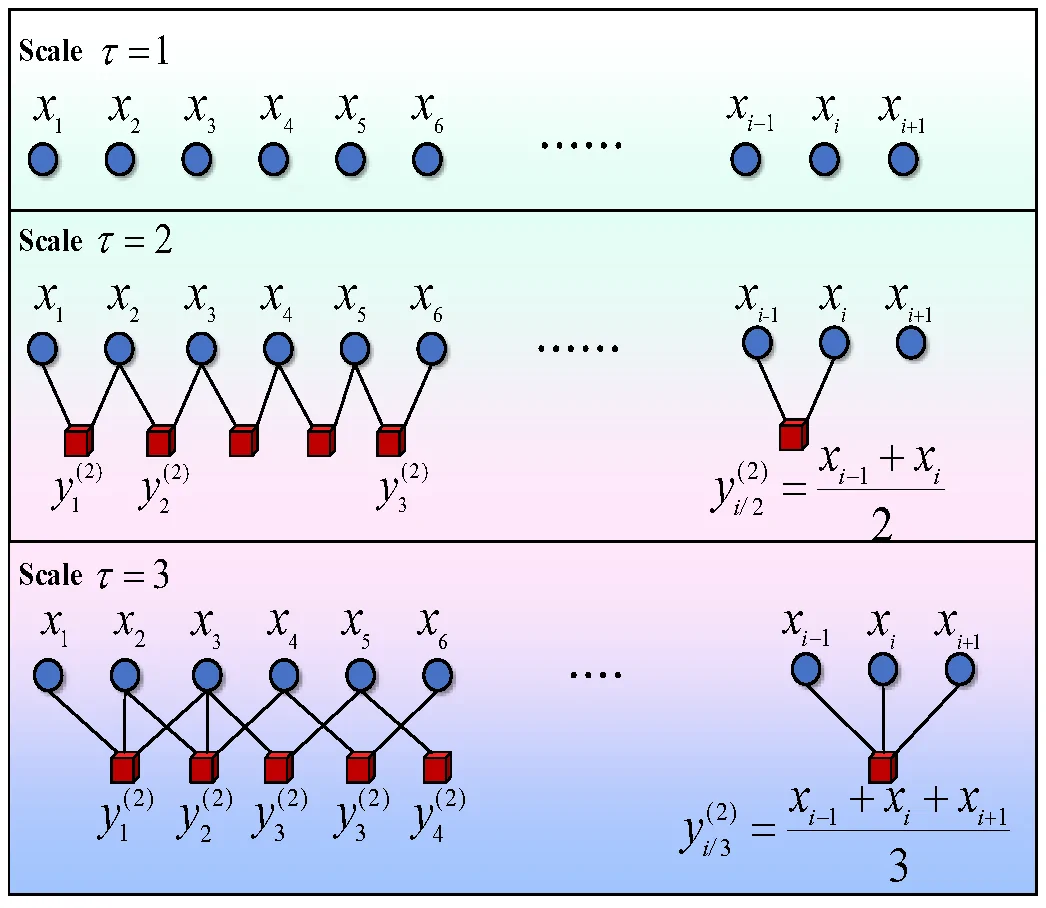
Moving-average coarse-graining in modified multi-scale entropy.

This operation produces a sequence of length N−τ+1, preserving more samples at large scales than standard coarse-graining. The moving-average construction is illustrated in Figure 4.

Entropy is then evaluated once on the elongated sequence:

(4) $${MME}_{t}\left(X,m,p,\tau \right)=E_{t}\left(y_{\mathrm{mod}}^{\left(\tau \right)},m,p\right)$$

Here, the coarse-grained sequence is generated by sliding averaging, but the downstream entropy operator is unchanged.

MME therefore modifies only the windowing rule, not the entropy core.

#### 2.1.3. Composite Multi-Scale Entropy

Composite multi-scale entropy (CME) stabilizes MSE at large scales by generating τ shifted coarse-grained sequences instead of a single path [25].

(5) $$y_{j}^{\left(\tau ,k\right)}=\frac{1}{\tau }\sum_{i=\left(j-1\right)\tau +k}^{j\tau +k-1}x_{i}$$

**Figure 5:**
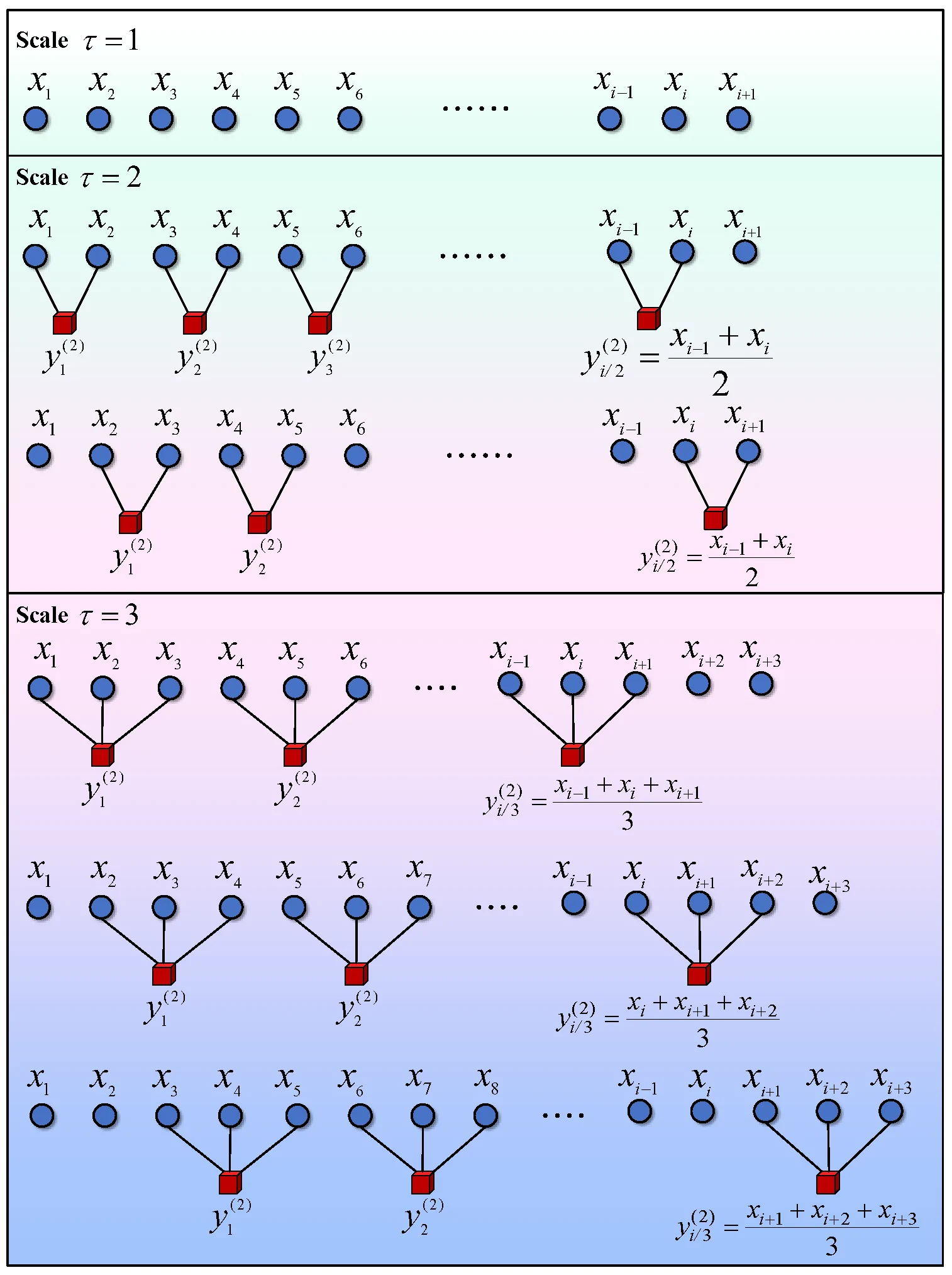
Shifted path coarse-graining in composite multi-scale entropy.

Each shifted sequence has length floor (N/τ). The shifted-path construction is illustrated in Figure 5.

The final CME value is the arithmetic mean of the τ entropy estimates, so CME changes only the path generation stage and preserves the original entropy operator.

The principle of the τ-shifted realizations is to avoid relying on only one coarse-graining phase. At a given scale, each shifted sequence starts from a different offset, so the collection of shifted paths samples different portions of the original signal. Although each path is shorter than the original signal, their joint use reduces phase dependence and improves the coverage of non-overlapping local regions across the full record.

(6) $${CME}_{t}\left(X,m,p,\tau \right)=\frac{1}{\tau }\sum_{k=1}^{\tau }E_{t}\left(y^{\left(\tau ,k\right)},m,p\right)$$

Here, $E_{t}$ denotes the corresponding single-scale entropy for t ∈ {DE, FE, PE}.

#### 2.1.4. Weighted, Composite, and Refined Variants

**Figure 6:**
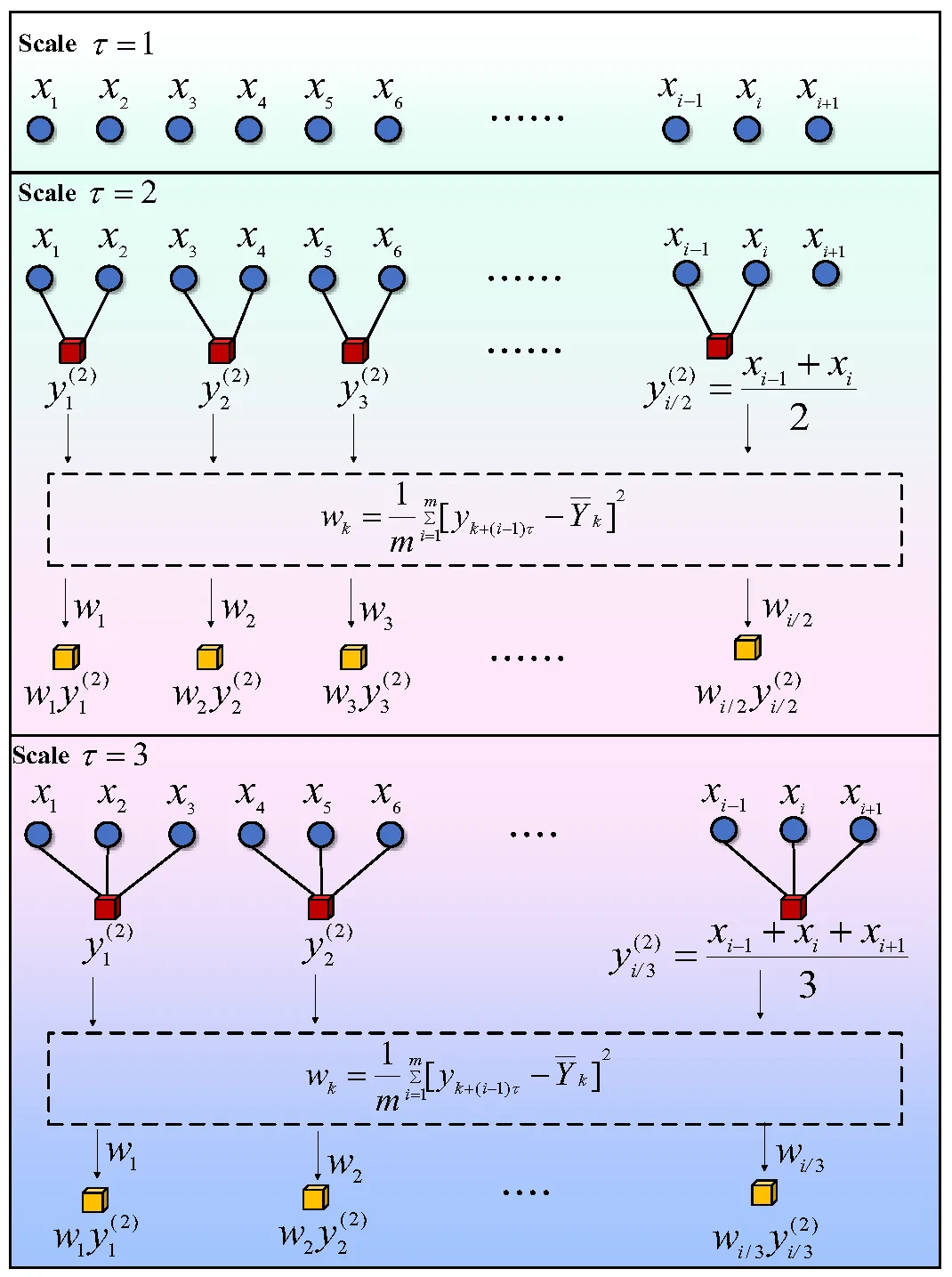
Representative weighting scheme for multi-scale weighted entropy.

The remaining extensions in the canonical family are most compactly understood as operator substitutions relative to MSE. Multi-scale weighted entropy (MWE) introduces an amplitude-sensitive weight w for each embedding vector, typically its variance [26,27]:

(7) $$w_{k}=\frac{1}{m}\sum_{i=1}^{m}\;[y_{k+\left(i-1\right)\tau }-{{\bar{Y}}_{k}]}^{2}$$

where ${\bar{Y}}_{k}$ is the mean of the embedding vector $\left\{y_{k}^{\tau },y_{k+\tau }^{\tau },\ldots ,y_{k+\left(m-1\right)\tau }^{\tau }\right\}$. The weighting process is illustrated in Figure 6.

MWE injects w into pattern probabilities or similarity statistics and therefore restores amplitude information that is suppressed by purely symbol-based encodings.

A representative weighted formulation is:

(8) $${MWE}_{t}\left(X,m,p,\tau \right)=E_{t,W}\left(y^{\left(\tau \right)},m,p,w\right)$$

Composite multi-scale weighted entropy (CMWE) applies the same weighted entropy after composite path generation; its difference from MWE lies only in replacing the single coarse-graining path with τ shifted paths [28].

Refined composite multi-scale entropy (RCME) changes the aggregation rule rather than the signal representation [29]: instead of averaging final entropy values, it first averages the intermediate statistical profiles and then applies the nonlinear entropy operator once. For PE and DE, the aggregated object is a pattern distribution; for FE, it is a similarity function.

**Figure 7:**
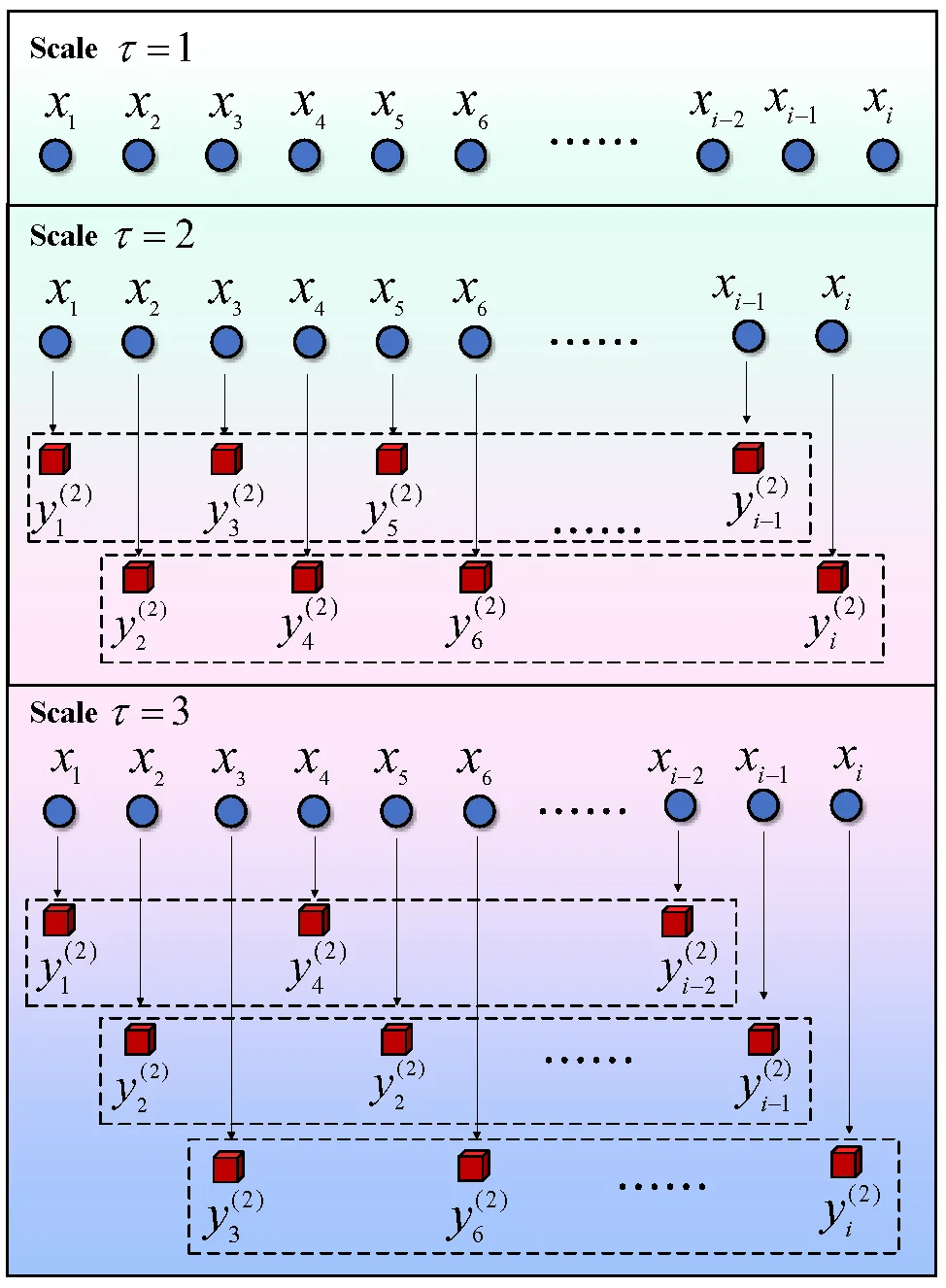
Representative downsampling scheme in RCDE.

Refined composite multi-scale weighted entropy (RCMWE) combines refined aggregation with amplitude weighting [30]. Refined composite multi-scale global fuzzy entropy (RCMGFE) is an FE-specific modification that uses global rather than local mean normalization [31], whereas refined composite downsampling entropy (RCDE) preserves refined aggregation but replaces averaging-based coarse-graining with direct downsampling [32]; Figure 7 shows the representative downsampling scheme.

Accordingly, the weighted/composite/refined branch differs from MSE along only three axes: path generation, statistical profile construction, and the point at which multi-path information is aggregated.

It is important to distinguish refined composite processing from simple downsampling. Simple downsampling may discard fault-related high-frequency or transient components, whereas refined composite methods use multiple shifted realizations and aggregate intermediate statistical profiles to stabilize entropy estimation. The term refined therefore refers to the aggregation and estimation strategy, not to an assumption that downsampling itself always preserves diagnostic information.

Most composite and refined composite methods use the arithmetic mean because it provides a simple and reproducible estimator across shifted paths. Median-, mode-, or frequency-based aggregation could be useful when entropy values are highly skewed or contain outliers, but such alternatives would define new methodological variants and require separate validation. They are therefore treated as future extensions rather than silently substituted into the published methods benchmarked here.

### 2.2. Generalized Multi-Scale Methods

Generalized multi-scale methods replace mean-only coarse-graining with moment-based coarse-graining so that multi-scale features can reflect not only local level but also volatility and asymmetry [33,34,35,36].

#### 2.2.1. Moment-Based Baseline

Generalized multi-scale entropy (GME) constructs a moment-specific sequence for each scale and then computes entropy on that sequence [36].

For a time series x, the first three moment-based sequences over non-overlapping windows of length τ correspond to the mean, variance, and the third central moment, respectively:

(1) First moment (mean):

(9) $$y_{1,j}^{\left(\tau \right)}=\frac{1}{\tau }\sum_{i=\left(j-1\right)\tau +1}^{j\tau }x_{i}$$

(2) Second moment (variance):

(10) $$y_{2,j}^{\left(\tau \right)}=\frac{1}{\tau }\sum_{i=\left(j-1\right)\tau +1}^{j\tau }{\left(x_{i}-y_{1,j}^{\left(\tau \right)}\right)}^{2}$$

(3) Third central moment:

(11) $$y_{3,j}^{\left(\tau \right)}=\frac{1}{\tau }\sum_{i=\left(j-1\right)\tau +1}^{j\tau }{\left(x_{i}-y_{1,j}^{\left(\tau \right)}\right)}^{3}$$

Entropy is computed on each moment-specific sequence, yielding distinct mean-, variance-, and third-central-moment-based multi-scale representations.

(12) $${\mathrm{GME}}_{t,n}(X,m,p,\tau )=E_{t}(y_{n}^{\left(\tau \right)},m,p)$$

Here, n ∈ {1,2,3} denotes the moment order and t ∈ {DE, FE, PE} denotes the entropy type.

#### 2.2.2. Composite and Refined Variants

GME defines the informative object in this family; GCME and GRCME only change how that object is estimated. GCME introduces τ shifted realizations for each selected moment and follows the same calculate-then-average rule as CME, thereby improving estimator stability at large scales.

GRCME keeps the same shifted realizations but averages the intermediate statistical profile before a single entropy evaluation. Within the generalized family, the substantive modeling choice is therefore the moment order, whereas composite and refined variants merely change the aggregation rule.

### 2.3. Fractional Multi-Scale Methods

Fractional multi-scale methods keep the multi-scale representation fixed but replace the conventional integer-order entropy operator with a fractional-order counterpart governed by α, thereby targeting nonlocal dependence and long-range memory [17,37,38,39]. Because the source literature introduced several specific realizations rather than a single generic template, the benchmark uses unified notation here to highlight their shared operator-level structure.

#### 2.3.1. Fractional Baseline

Representative fractional realizations retain standard coarse-graining and change only the final entropy evaluation [17].

(13) $${\mathrm{MFrE}}_{t}(X,m,p,\tau ,\alpha )={\mathrm{FE}}_{t}(y^{\left(\tau \right)},m,p,\alpha )$$

Here, FE_t(·, α) denotes the fractional-order extension of E_t, rather than fuzzy entropy. For PE and DE, the fractional operator acts on a pattern-probability distribution; for FE, it acts on the similarity-ratio term. The common purpose is to alter the entropy operator without changing the multi-scale signal generation stage.

#### 2.3.2. Refined, Weighted, and Phase-Domain Variants

Refined composite multi-scale fractional entropy (RCMFrE) applies the same fractional operator to the refined statistical profile used by RCME, thereby combining estimator stabilization with fractional-order evaluation. The representative source paper introduced an FE-based realization [37], and the benchmark adopts unified notation to express the corresponding compatible variants.

When α = 0, this construction reduces to the corresponding integer-order RCME.

Multi-scale weighted fractional entropy (MWFrE) adds amplitude weighting before fractional evaluation. Representative source implementations are permutation-based [17], whereas the benchmark applies the same operator logic to other compatible back-ends for controlled comparison.

Its difference from MWE is therefore not the weighted profile itself but the fractional operator applied to that profile.

Fractional multi-scale phase entropy (FMPE) changes the signal domain rather than the operator architecture. The original series is first converted into an instantaneous phase sequence via the Hilbert transform, after which composite multi-scale processing and weighted fractional entropy are applied. The source literature introduced a phase-permutation realization [39]; the benchmark uses the same phase-domain front-end with compatible FE and DE back-ends for comparison.

Across the fractional family, the key design choice is the statistical object supplied to the same fractional operator: raw, refined, weighted, or phase-derived.

### 2.4. Hierarchical Methods

Conventional coarse-graining acts as a low-pass filter and progressively suppresses high-frequency components [18,40]. Hierarchical methods address this limitation by recursively separating a signal into low-frequency and high-frequency components, thereby retaining information across the spectrum.

#### 2.4.1. Hierarchical Entropy

Hierarchical entropy (HE) replaces coarse-graining with recursive signal layering [18].

At each layer, HE applies an averaging operator to extract low-frequency trends and a difference operator to preserve high-frequency details. These operators are equivalent to one decomposition step of the Haar wavelet transform, which explains the full-spectrum character of the resulting representation. The use of Haar-equivalent operators is mainly motivated by their dyadic structure, low computational cost, and direct approximation/detail separation. Other wavelet bases may capture smoother or more oscillatory structures, but they introduce additional basis-selection choices and are therefore better treated as method-specific extensions rather than as a universal replacement in this benchmark.

Iterating the two operators yields a binary tree of hierarchical components $X_{\left\{l,e\right\}}$, where l is the layer index and e is the node index.

For a time series $x=\{x_{i},i=1,2,\ldots ,N\}$, the operators are defined as:

(14) $$Q_{0}\left(x\right)=\frac{x_{2i+1}+x_{2i+2}}{2},Q_{1}\left(x\right)=\frac{x_{2i+1}-x_{2i+2}}{2}$$

A single-scale entropy measure is then computed for each hierarchical component, and the resulting values form the HE feature vector:

(15) $$HE_{t}\left(X,l,e,m,p\right)=E_{t}\left(X_{l,e},m,p\right)$$

**Figure 8:**
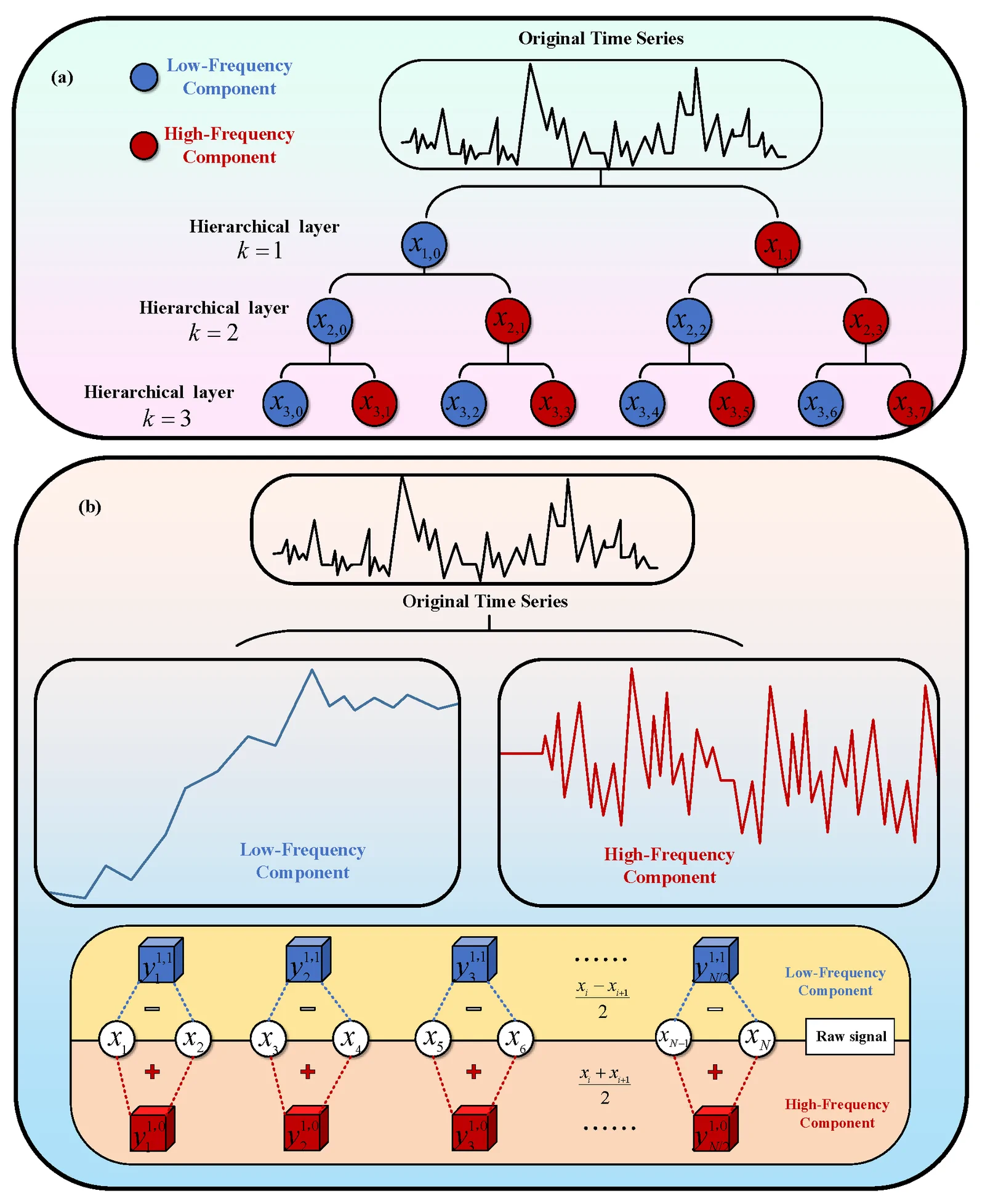
Operator-driven layering in hierarchical entropy: (**a**) recursive hierarchical decomposition into low-frequency (blue) and high-frequency (red) components; (**b**) averaging and difference operations used to generate the two components from paired samples.

Here, t ∈{DE, FE, PE} and $X_{\left\{l,e\right\}}$ denotes the component at layer l and node e. The hierarchical decomposition and the associated averaging and difference operators are illustrated in Figure 8.

#### 2.4.2. Hierarchical Variants

Hierarchical multi-scale entropy (HME) extends HE by coarse-graining each hierarchical component across time, thereby forming a joint frequency-band/time-scale representation [41,42].

Modified hierarchical entropy (MHE) changes the layering operator rather than the entropy definition: it replaces non-overlapping averaging and differencing with sliding operators, which preserves more samples and removes the power-of-two constraint [40,43].

Modified hierarchical amplitude-aware permutation entropy (MHAAPE) keeps the MHE decomposition but replaces PE with amplitude-aware permutation entropy, making the hierarchical feature sensitive to both ordinal structure and amplitude variation [43,44,45,46,47].

Two-dimensional multi-scale hierarchical time–frequency methods (MHTF-PE2D and MHTF-FE2D) move the selected hierarchical components to 2D time–frequency maps and evaluate their complexity in the joint time–frequency domain. Representative source papers introduced permutation- and dispersion-based realizations [48,49], whereas the benchmark evaluates the same hierarchical time–frequency front-end with compatible entropy back-ends, including FE2D. Only low-frequency branches are further decomposed, which keeps the feature map focused on physically meaningful time–frequency structure.

Accordingly, hierarchical variants differ mainly in component generation (non-overlapping vs. sliding), the entropy core (standard vs. amplitude-aware), and the representation domain (1D band signals vs. 2D time–frequency maps).

### 2.5. Additional Variants

The remaining methods are structurally more heterogeneous than the canonical, generalized, fractional, and hierarchical families described above. Their extensions mainly follow four directions: preprocessing or reconstructing the sequence supplied to entropy evaluation, redefining the dynamic structure used for complexity characterization, enriching the multi-scale representation through distribution-aware or wavelet packet-based constructions, and expanding frequency-domain coverage through multi-basis decomposition. Most methods in this group were introduced with a specific entropy back-end. When the front-end transformation was transferable, it was paired with compatible entropy operators in the benchmark for controlled comparison.

#### 2.5.1. Preprocessing- and Reconstruction-Based Variants

This group modifies the input sequence before multi-scale entropy evaluation. Representative strategies include adaptive signal reconstruction and interpolation-assisted multi-scale generation.

Multi-scale inherent entropy (MIE) applies adaptive decomposition before multi-scale analysis to reduce the influence of noise and non-stationarity [50]. Methods such as empirical mode decomposition (EMD) or partly ensemble local characteristic-scale decomposition (PELCD) first separate oscillatory components. Selected components are then recombined to construct an inherent signal that retains the dominant dynamic structure. Multi-scale entropy is computed from the reconstructed signal rather than from the raw sequence:

(16) $$MIE_{t}\left(X,m,p,\tau \right)=E_{t}\left(y_{inherent}^{\left(\tau \right)},m,p\right)$$

Here, $y_{inherent}^{\left(\tau \right)}$ is the coarse-grained sequence obtained from the reconstructed inherent signal.

Composite interpolation multi-scale entropy (CIME) combines two parallel multi-scale constructions: conventional composite coarse-graining and interpolation-based multi-scale generation [51]. The composite branch preserves the stabilizing effect of shifted coarse-grained sequences, whereas the interpolation branch retains nonlinear local structure that may be attenuated by averaging-based coarse-graining. The final CIME value is obtained by combining the two entropy components:

(17) $$CIME_{t\left(X,m,p,\tau \right)}=\frac{1}{2\left[CME_{t\left(X,m,p,\tau \right)}+IME_{t\left(X,m,p,\tau \right)}\right]}$$

Here, $IME_{t}$ denotes the entropy computed from the interpolation-generated series.

MIE and CIME differ in their preprocessing logic. MIE reconstructs an inherent signal before multi-scale entropy analysis, whereas CIME augments conventional composite processing with an interpolation-derived multi-scale branch.

For interpolation-based multi-scale generation, realism depends on whether the interpolation operation preserves the local temporal structure of the vibration signal. In this benchmark, interpolation is used only as a transformation inside published multi-scale entropy variants; it is not used to create new fault labels or artificially expand the dataset. Such methods should be applied cautiously when impulsive transients or sparse fault signatures are dominant, because interpolation may smooth or reshape diagnostically meaningful events.

#### 2.5.2. Transition-, Derivative-, and Graph-Based Variants

This group changes the object on which entropy is evaluated. Instead of characterizing complexity only from coarse-grained amplitude sequences, these methods use symbolic transitions, successive derivatives, or graph-based relational structures.

Composite multi-scale transition entropy (CMTE) measures the evolution of symbolic states rather than only their marginal frequencies [52]. For each composite path, a transition probability matrix is constructed from the symbolic sequence, and entropy is computed from its positive eigenvalues. CMTE retains the calculate-then-average structure of composite multi-scale analysis:

(18) $$CMTE_{t}\left(X,m,p,\tau \right)=\frac{1}{\tau }\sum_{k=1}^{\tau }TE_{t}\left(y_{k}^{\left(\tau \right)},m,p\right)$$

Here, $TE_{t}$ denotes the entropy computed from the eigenvalues of the transition matrix built from sequence $y_{k}^{\left(\tau \right)}$.

Derivative multi-scale entropy (DMS-Entropy) replaces temporal coarse-graining with successive discrete derivatives [53]. It characterizes signal dynamics through multiple orders of temporal variation, including first-order changes and higher-order difference structures. For a time series x(t), the first derivative is defined as x′(t)=x(t+1)−x(t), and higher-order derivatives are generated recursively. The entropy values obtained from derivative sequences of different orders are concatenated into the final feature vector:

(19) $${DME}_{t}(X,D,m,p)=[E_{t}\left(x^{\left(n\right)},m,p\right){]}_{n=1}^{D}$$

Here, $x^{\left(n\right)}$ is the n-th derivative sequence and D is the maximum derivative order.

Graph multi-scale permutation entropy (MPEG) transforms the original time series into a horizontal visibility graph (HVG) so that temporal and topological relations can be analyzed jointly [54]. Multi-scale processing is performed through graph coarsening, and ordinal patterns are constructed from graph-based neighborhood structures. The final entropy is computed from the probability distribution of graph-derived permutation patterns:

(20) $$MPEG\left(\tau \right)=-\sum_{k=1}^{K}p_{k}^{\left(\tau \right)}\log p_{k}^{\left(\tau \right)}$$

where $p_{k}^{\tau }$ denotes the probability of the k-th graph-derived ordinal pattern at scale τ, and K is the number of distinct patterns. Because MPEG is intrinsically permutation-based, only the PE realization was included in the benchmark.

These variants modify the representation used for entropy evaluation. CMTE encodes symbolic transition dynamics, DMS-Entropy encodes multi-order temporal variations, and MPEG encodes graph-based relational structures.

#### 2.5.3. Distribution-Aware and Wavelet Packet-Based Variants

This group enriches the multi-scale representation before entropy calculation. The corresponding strategies include adaptive distribution modeling, improved symbolization, wavelet packet-based fine-to-coarse reconstruction, adaptive sub-signal selection, and frequency-band-specific zoom processing.

Multi-scale generalized Gaussian distribution improved entropy (MGGDIE) combines adaptive distribution normalization with information-preserving symbolization [55]. A generalized Gaussian distribution (GGD) normalizes each coarse-grained sequence, enabling the transformation to adapt to different empirical signal distributions. The subsequent symbolization step retains both absolute amplitude information and local temporal variation, rather than relying only on ordinal relationships. The resulting entropy can be expressed as:

(21) $${MGGDIE}_{t}\left(X,m,L,\tau ,\beta \right)={IE}_{t}\left(GGD\left(y^{\left(\tau \right)},\beta \right),m,L\right)$$

Here, t ∈ {DE, PE}, β is the GGD shape parameter, and ${IE}_{t}$ denotes the entropy of the adaptively normalized and symbolized sequence. Here, L is the number of symbols used for sequence symbolization.

Fine-to-coarse multi-scale entropy (F2CME) constructs multi-scale sequences through wavelet packet decomposition (WPD) [56]. The original signal is decomposed into frequency-band sub-signals, and a nested sequence of reconstructions is generated by progressively removing high-frequency components.

This procedure creates fine-to-coarse representations while preserving the original sequence length. The entropy evaluation can be written as:

(22) $$F2CME_{t\left(X,j,m,p,\tau \right)}=E_{t\left(F2C^{\tau },m,p\right)}$$

where $F2C(\tau )=\sum_{i=0}^{2^{j-1}-\tau }R_{j,i}$ is the multi-scale time series at scale τ, with $R_{j,i}$ being the reconstructed sub-signals from a j-level WPD.

**Figure 9:**
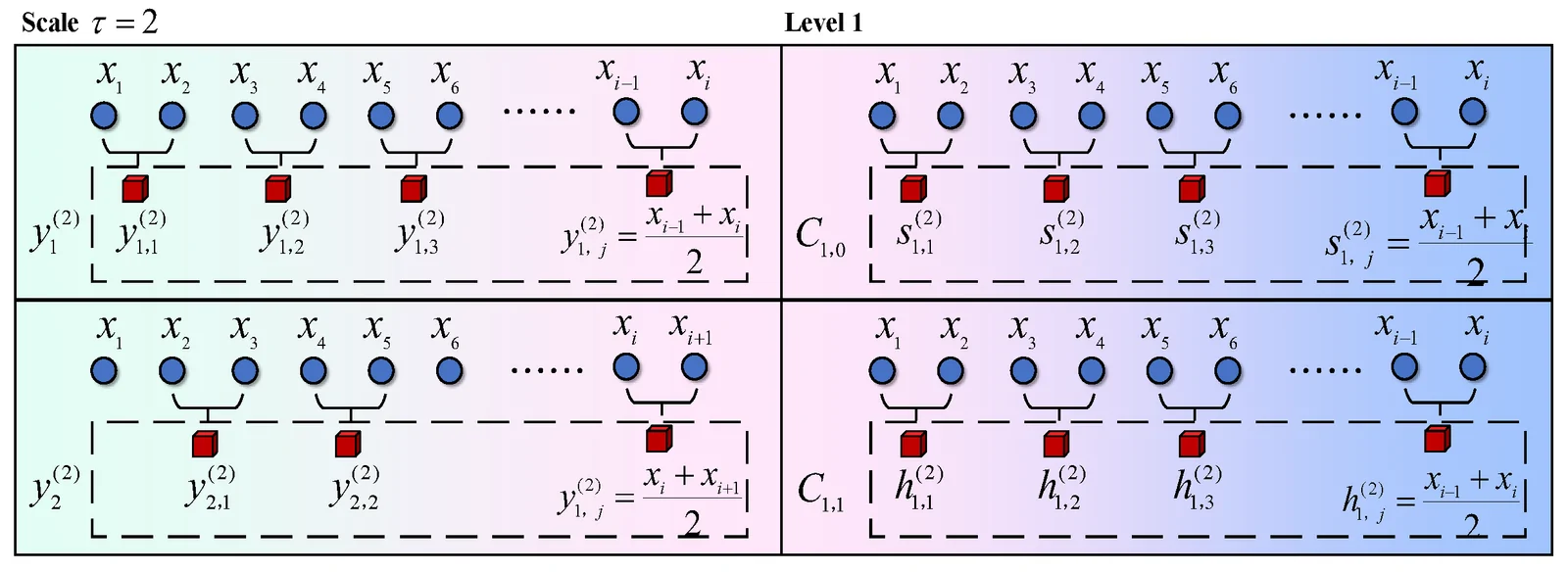
Fine-to-coarse signal generation process.

Adaptive multi-scale weighted entropy (AMWE) extends the F2CME framework by adding adaptive sub-signal selection and amplitude-sensitive entropy evaluation [57]. Wavelet packet sub-signals are first ranked according to their similarity to the original sequence, and the selected components are used to form adaptive fine-to-coarse representations. Weighted entropy is then evaluated on the resulting sequences:

(23) $$AMWE_{t\left(X,j,m,p,\tau \right)}=E_{\left(t,W\right)\left(F2C_{adaptive}^{\tau },m,p\right)}$$

where ${F2C}_{\mathrm{adaptive}}^{\left(\tau \right)}=\sum_{i=\tau }^{n-\tau +1}U_{i}$ is the multi-scale series at scale τ, generated from the adaptively selected subset of n sub-signals {$U_{i}$}. The fine-to-coarse signal-generation process is illustrated in Figure 9.

Refined composite zoom multi-scale weighted entropy (RCZMWE) adopts a decomposition-first strategy that processes individual frequency-band subsequences rather than cumulative reconstructions [58]. The raw signal is first decomposed by WPD into band-limited subsequences. Each subsequence then undergoes zoom coarse-graining, and a weighted entropy value is computed for every generated zoom sequence. The final method output is obtained by averaging these entropy values:

(24) $$RCZMWE_{t\left(X,\xi ,c,m,p,\tau \right)}=\frac{1}{N_{s}\sum_{k=1}^{N_{s}}E_{\left(t,W\right)\left(Y_{k},m,p\right)}}$$

where $N_{s}$ is the total number of multi-scale sequences generated, and $E_{t,W}$ is the weighted entropy function for type t ∈ {DE,PE}. Here, ξ denotes the selected wavelet basis and c is the wavelet-packet decomposition level.

These methods share a representation-enrichment strategy, but they operate at different stages. MGGDIE modifies statistical normalization and symbolization, F2CME constructs fine-to-coarse reconstructions, AMWE adds adaptive sub-signal selection and weighting, and RCZMWE performs band-specific zoom processing before weighted entropy aggregation.

#### 2.5.4. Concentric Entropy

Concentric entropy (CE) denotes a multi-basis decomposition framework abstracted from concentric diversity entropy [59]. The original literature introduced a DE-based realization; the benchmark applies the same multi-basis front-end to compatible PE and FE back-ends for controlled comparison.

CE decomposes the signal in parallel with multiple wavelet families, such as Haar, Symlets, and Biorthogonal wavelets.

**Figure 10:**
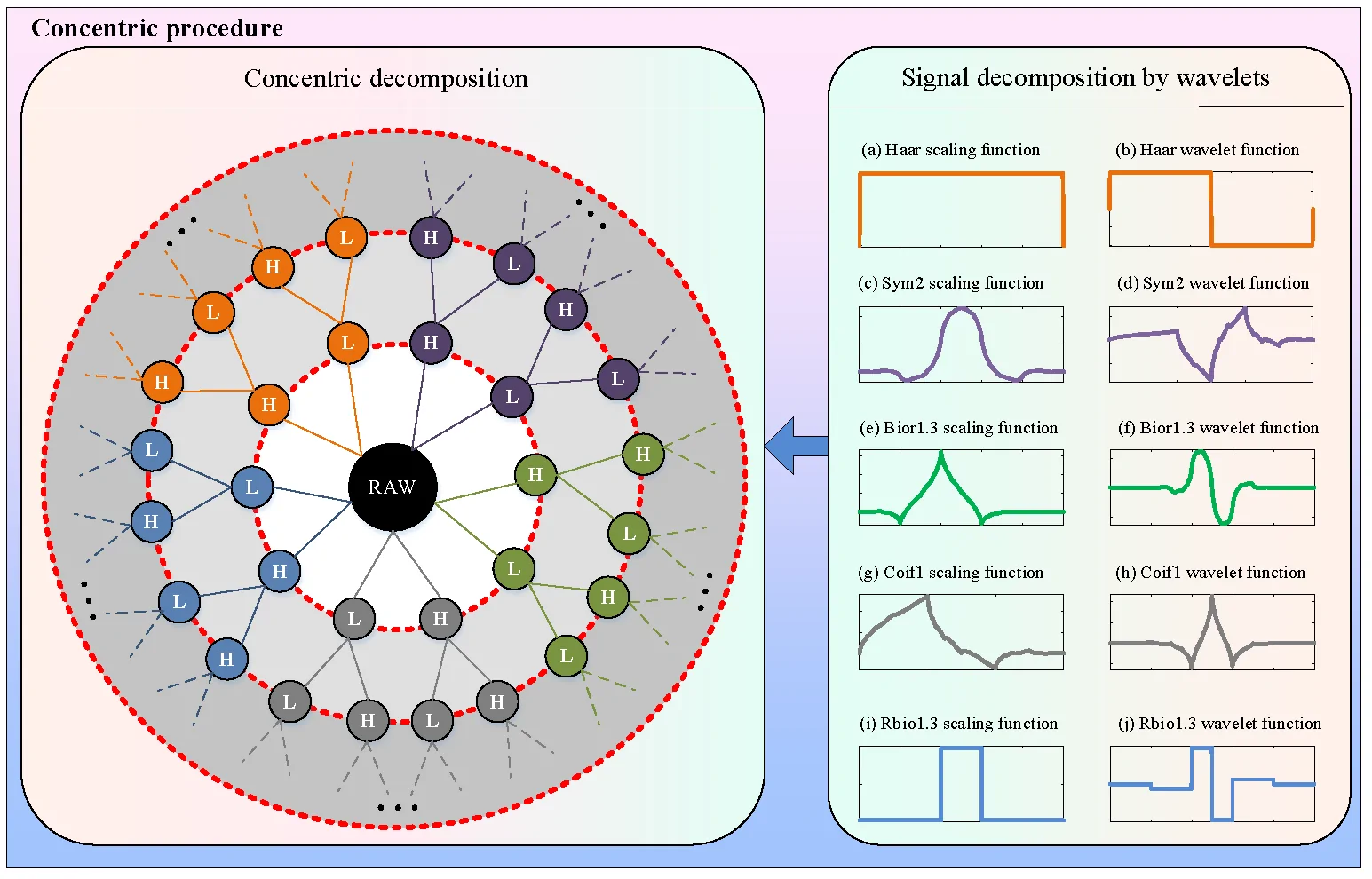
Parallel multi-basis decomposition in concentric entropy. (Different colors distinguish the parallel basis-specific decomposition branches and do not represent numerical magnitude).

For each basis, recursive low-pass and high-pass filtering generates a tree of local refinement time series (LRTS). This process is structurally analogous to hierarchical entropy, but is repeated across multiple bases rather than a single one. The parallel multi-basis decomposition is illustrated in Figure 10.

(25) $$Y_{2n}^{r,w}=\left(↓2\right)\left(H_{w}*Y_{n}^{r-1,w}\right)$$

(26) $$Y_{2n+1}^{r,w}=\left(↓2\right)\left(G_{w}*Y_{n}^{r-1,w}\right)$$

Here, * denotes convolution and ↓2 denotes decimation (downsampling). The term concentric refers to the parallel multi-basis decomposition.

Entropy is computed for each LRTS individually, and the resulting values are concatenated to form the final multi-basis feature vector:

(27) $$CE_{t\left(X,m,p,W\right)}={\left[E_{t\left(Y_{i}^{r,w},m,p\right)}\right]}_{\left(r,w,i\right)}$$

Here, W is the set of selected wavelet bases and $E_{t}$ is the single-scale entropy function. Here, r is the decomposition level, w is a selected wavelet basis, and i is the node index. The components indexed by (r, w, i) are concatenated to form the final multi-basis feature vector.

## 3. Experimental Setup

### 3.1. Dataset

**Figure 11:**
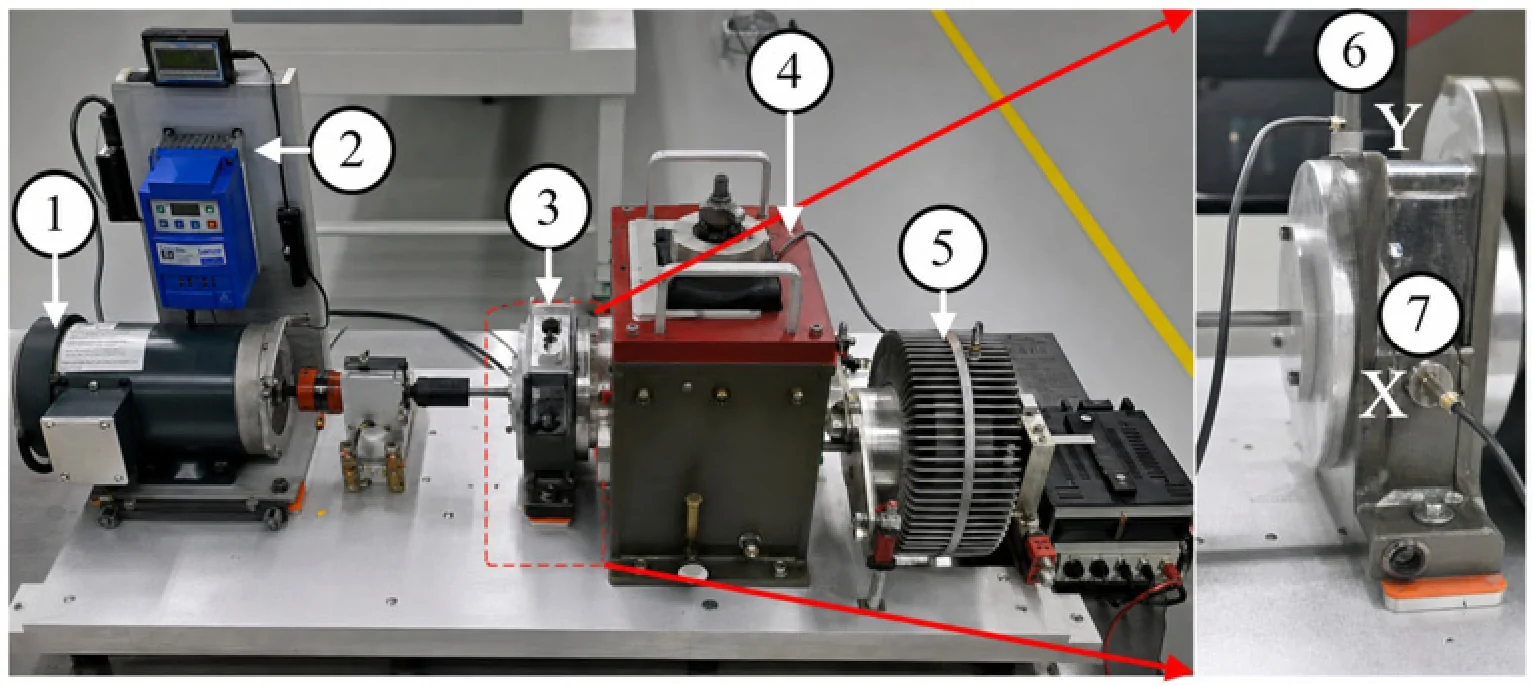
Modular test rig (1: motor; 2: controller; 3: planetary gearbox; 4: parallel gearbox; 5: brake; 6 and 7: accelerometers; X and Y indicate the measurement directions).

The benchmark uses the public XJTU-Gearbox dataset [60]. The test rig consists of a planetary gearbox driven by a 3-phase, 3 HP AC motor and controlled by a programmable logic controller. Vibration was recorded using PCB 352C04 accelerometers (PCB Piezotronics, Inc., Depew, NY, USA) mounted in the X and Y directions; only the Y-direction signal was used here to keep the comparison single-channel and uniform across all methods. The sampling frequency was 20,480 Hz and the motor speed was fixed at 1800 rpm. The modular test rig is shown in Figure 11.

**Figure 12:**
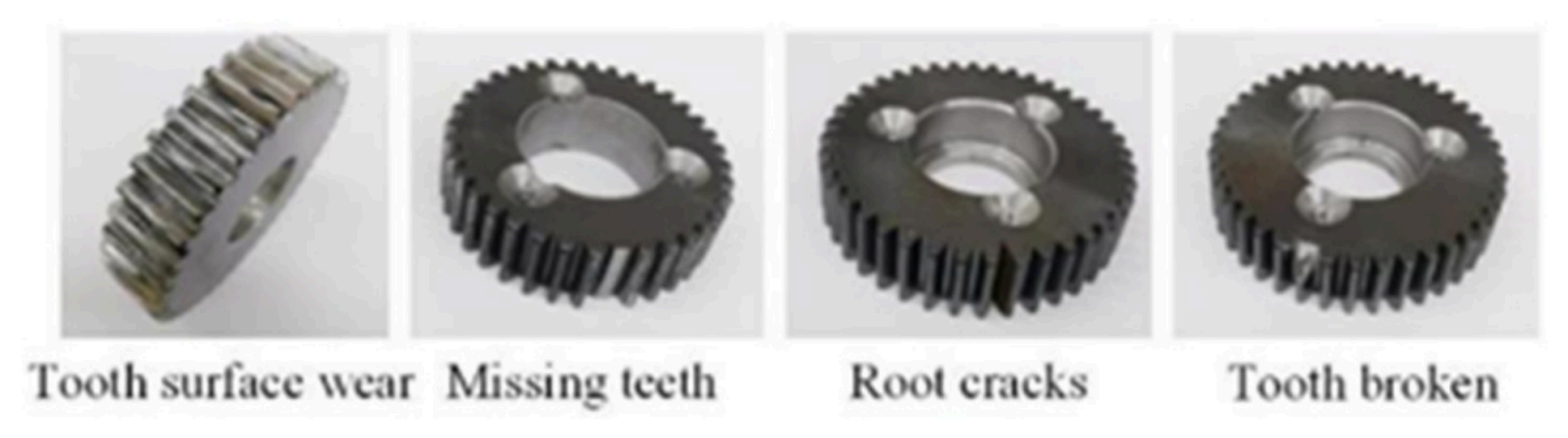
Gear fault examples in the XJTU-Gearbox dataset.

The dataset contains one healthy condition and four gear fault types, as shown in Figure 12: tooth surface wear, missing tooth, root crack, and broken tooth.

### 3.2. Experimental Protocol

A total of 85 multi-scale entropy feature sets were implemented, spanning permutation, fuzzy, and diversity entropy families and covering original, composite, refined, generalized, hierarchical, and several additional variants.

No feature selection or dimensionality reduction was applied; each method used its full multi-scale feature vector. To emphasize feature quality rather than classifier complexity, classification was performed using a k-nearest neighbors classifier with k = 5 and Euclidean distance. This deliberately simple and fixed classifier reduces the influence of classifier-specific hyperparameter tuning, so that the reported differences primarily reflect entropy feature quality. The data were randomly split into 70% training and 30% testing sets, and each experiment was repeated 20 times. Mean accuracy, standard deviation, and feature extraction time under a unified implementation workflow were recorded. No distance weighting or classifier hyperparameter tuning was used.

### 3.3. Parameter Settings and Fairness of Comparison

For fairness, the global experimental protocol was kept identical for all entropy methods, including the dataset, signal channel, segment length, number of samples per class, train/test split, classifier, repeated-run protocol, and evaluation metrics. The internal parameters of PE-, FE-, and DE-based methods were not forced to be numerically identical because they have different mathematical meanings. Instead, method-specific parameters followed the original papers, common practice, or the available author/benchmark code, and no method family was individually tuned on the test set. The common and method-specific parameter settings are summarized in Table 1.

## 4. Results and Discussion

Table 2 summarizes the benchmark results for 85 multi-scale entropy implementations by grouping PE-, FE-, and DE-based variants under their shared method frameworks. The following analysis focuses on the algorithmic mechanisms that most strongly shape diagnostic accuracy and computational cost.

Overall, the benchmark shows that diagnostic accuracy is not monotonically related to algorithmic complexity. Several computationally intensive methods fail to achieve competitive accuracy, whereas some comparatively simple representation-enriching methods attain both high accuracy and low runtime. The decisive factor is therefore not the number of processing stages, but whether the multi-scale transformation preserves fault-relevant temporal, amplitude, and frequency-domain information.

### 4.1. Statistical Interpretation of Close Differences

Because many methods differ by only 1–3 percentage points, numerical ranking alone should be interpreted cautiously. The 20 repeated runs provide a basis for estimating variability; therefore, methods with overlapping mean +/− standard deviation ranges are not treated as decisively different unless supported by paired run-level statistical evidence. When complete paired run-level records are available, a Friedman test followed by post hoc Wilcoxon signed-rank tests with multiple-comparison correction or Nemenyi-style analysis should be used to verify whether the top-ranked methods differ significantly. In the absence of complete paired records for every implementation, the discussion emphasizes robust patterns and accuracy–cost trade-offs rather than over-interpreting marginal rank differences.

The computational cost reported in Table 2 should also be read together with algorithmic structure. Runtime increases with the number of scales, embedding dimension, shifted paths, refined composite aggregation, wavelet or hierarchical decomposition, graph construction, interpolation, and two-dimensional transformations. Consequently, an expensive method is not necessarily more informative; it is useful only when the added transformation preserves fault-relevant temporal, amplitude, or frequency-domain information.

### 4.2. Statistical Robustness

Statistical robustness is central to multi-scale entropy because entropy estimates become unreliable as coarse-grained sequences shorten. This subsection compares the original, composite, modified, and refined composite strategies to show how data utilization and estimator stability affect performance.

#### 4.2.1. Limitations of the Original Multi-Scale Methods

The original multi-scale methods—MPE, MFE, and MDE—serve as the benchmark baselines. Their mean accuracies are 53.20%, 62.63%, and 67.70%, respectively.

A key limitation of these baselines is sequence shortening: at scale τ, coarse-graining reduces the available samples to N/τ. As τ increases, entropy estimates become less stable and pattern counts decrease, which weakens class separability. Within this baseline group, MDE outperformed MPE and MFE, suggesting that its symbolization strategy is comparatively well matched to the present diagnostic task.

#### 4.2.2. Composite vs. Modified Multi-Scale Strategies

Two main strategies have been proposed to alleviate data sparsity: composite multi-scale entropy (CME), which averages across multiple coarse-graining paths, and modified multi-scale entropy (MME), which uses a sliding window to preserve more samples.

CME improves accuracy consistently across all three entropy types. Relative to the original methods, CMPE, CMFE, and CMDE improve by 9.93%, 10.14%, and 10.63%, respectively. This pattern indicates that more stable estimation improves feature quality, although the gain comes at the cost of repeated entropy evaluations at each scale.

MME generates a longer sequence of length N−τ+1, but its gains are smaller. The likely reason is that overlapping windows introduce highly correlated samples and therefore contribute less new discriminative information than the composite strategy. Its runtime also depends strongly on the entropy core: FE becomes much slower because it requires pairwise vector comparisons, whereas PE is affected far less. The practical value of MME is therefore strongly metric dependent.

#### 4.2.3. Advantage of Refined Composite Methods

RCME further improves statistical robustness by averaging intermediate statistics before applying the nonlinear entropy operator. In Table 2, RCMPE, RCMFE, and RCMDE reach 70.43%, 73.70%, and 79.47%, all above their composite counterparts, with the strongest relative gain appearing in PE.

The improvement can be traced to the point at which multi-path information is aggregated. CME averages final entropy values, whereas RCME first averages the underlying distributions or similarity profiles and then evaluates entropy once. Because entropy estimation involves nonlinear transformations, stabilizing the input before the nonlinear step can reduce estimation variance and bias. The smaller gain from CME to RCME than from MSE to CME also indicates diminishing returns: composite processing addresses the first-order data-shortening problem, whereas refined composite processing mainly improves the quality of the estimate.

### 4.3. Compatibility of Enhancement Strategies

Another major design issue is whether an auxiliary mechanism contributes complementary information or merely perturbs information that the base entropy already encodes. Variance-based weighting provides the clearest example [26,27].

The benchmark shows that the effect of weighting is back-end-specific rather than uniformly beneficial across entropy types.

#### 4.3.1. Weighting Mainly Benefits PE

For PE, variance-based weighting yields consistent gains. MWPE exceeds MPE by 3.57%, CMWPE improves on CMPE by 5.50%, and RCMWPE improves on RCMPE by 2.74%.

This pattern is plausible because PE is based on ordinal ranking and therefore discards absolute amplitude information. Weighting reintroduces part of the physical information carried by high-energy impacts and abrupt vibration responses, allowing patterns with similar order but very different amplitudes to be distinguished more clearly.

#### 4.3.2. DE and FE Reveal the Limits of Weighting

The same enhancement does not generalize to DE or FE in this benchmark. For DE, MWDE, CMWDE, and RCMWDE are all lower than their unweighted counterparts by 7.13%, 10.23%, and 14.20%, respectively. For FE, the degradation is likewise substantial: MWFE is 18.60% below MFE and RCMWFE is 15.17% below RCMFE.

The difference arises from compatibility with the base statistic. DE already retains part of the signal-amplitude distribution through class-based quantization, so extra variance weighting can overemphasize local fluctuations without adding genuinely new discriminative content. FE goes further: because it is built from distances between embedded vectors, amplitude sensitivity is already intrinsic to the entropy core. Additional weighting can therefore distort rather than enrich the similarity structure.

These results suggest a general design rule: enhancement mechanisms should be judged by complementarity, not novelty. A modification is useful only when it contributes information that the base entropy does not already encode. These observations support a complementarity-versus-redundancy principle: an enhancement is beneficial only when it supplies information not already encoded by the base entropy measure.

### 4.4. Hierarchical and Related Methods

Hierarchical methods address a different limitation of conventional multi-scale analysis: the loss of high-frequency information. Instead of averaging away these components, they separate the signal into low- and high-frequency parts and analyze both.

This shift from information suppression to information retention underlies the strong performance of HE-derived methods in the benchmark.

#### 4.4.1. Hierarchical Decomposition and High-Frequency Information

Hierarchical methods perform strongly overall. In particular, HMPE reaches 81.60%, outperforming all conventional coarse-graining-based PE variants.

Their core advantage is the recursive decomposition of each signal layer into an averaged component and a difference component. Unlike standard coarse-graining, which behaves as a low-pass filter, this procedure preserves high-frequency fault information. The results imply that discriminative fault information is not confined to low-frequency trends, but is distributed across the spectrum, especially in high-frequency components associated with transients and impacts.

**Figure 13:**
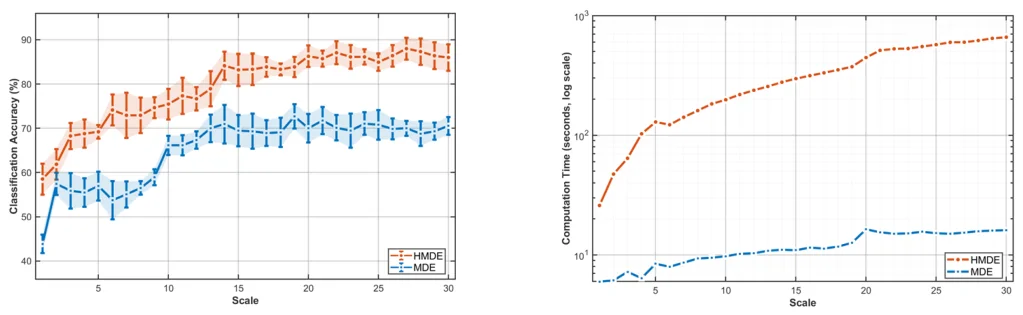
HMDE versus MDE across scales: (**left**) classification accuracy (mean ± SD); (**right**) computation time on a logarithmic scale.

**Figure 14:**
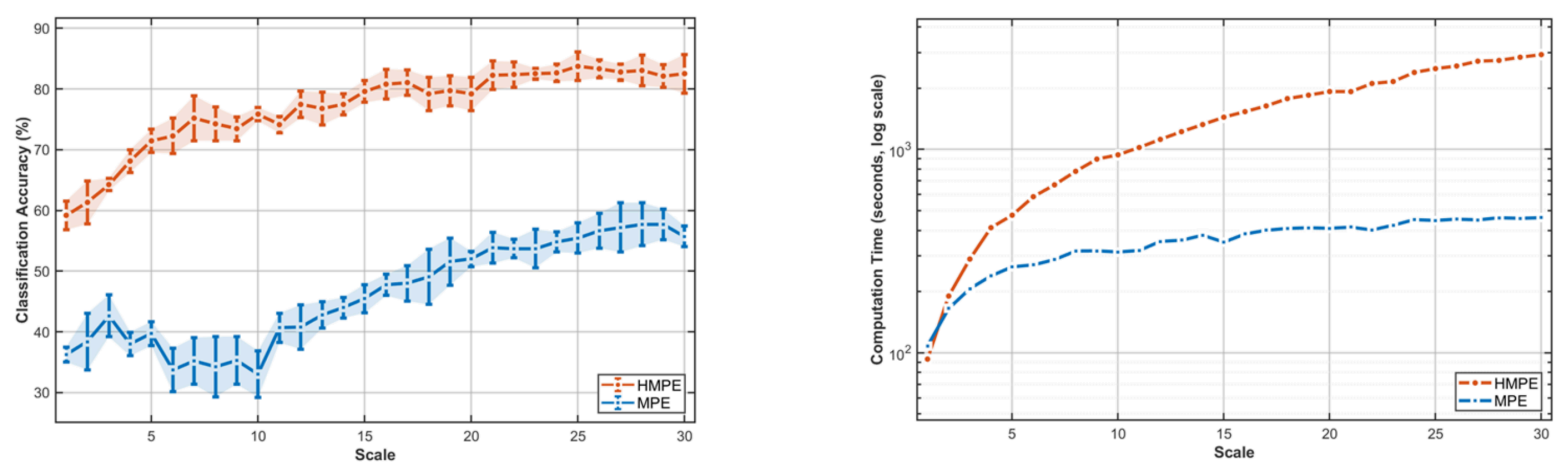
HMPE versus MPE across scales: (**left**) classification accuracy (mean ± SD); (**right**) computation time on a logarithmic scale.

**Figure 15:**
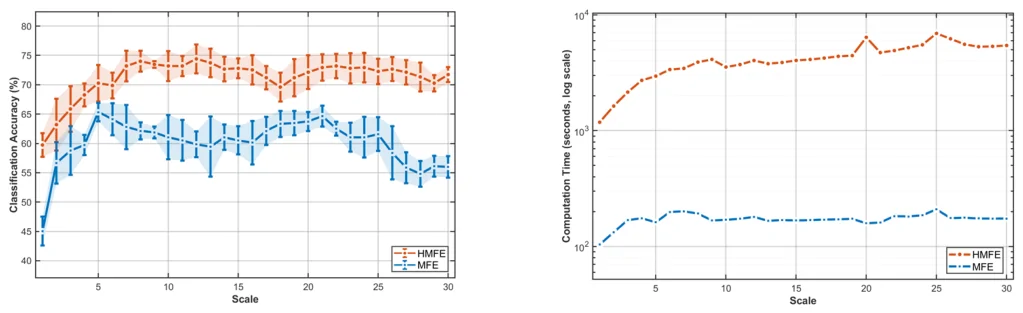
HMFE versus MFE across scales: (**left**) classification accuracy (mean ± SD); (**right**) computation time on a logarithmic scale.

This interpretation is supported by Figure 13, Figure 14 and Figure 15, where HMDE, HMPE, and HMFE consistently outperform their standard multi-scale counterparts over 30 scales.

The contrast is structural rather than merely statistical. Standard multi-scale methods first lose part of the signal spectrum through coarse-graining and then seek to stabilize the remaining representation through averaging. Hierarchical methods instead retain a broader portion of the original spectrum before entropy is computed, which better preserves transient and impact-related fault information.

The curves also reveal an information saturation point: accuracy rises rapidly at first, then plateaus and may decline as additional layers are introduced. Early layers separate frequency bands carrying dominant fault impacts and rotational components, whereas deeper layers may contribute redundant or noise-dominated components.

From a practical standpoint, this means that computing the full hierarchical tree is unnecessary. Truncating the decomposition near the saturation point can preserve near-optimal accuracy while reducing computational cost.

#### 4.4.2. CDE as a High-Efficiency Method

Concentric diversity entropy (CDE) achieves the strongest overall accuracy–efficiency trade-off in the benchmark, reaching 83.47% in only 0.62 min.

Its strength lies in parallel decomposition with multiple wavelet bases. Because different fault signatures are better represented by different bases, multi-basis analysis increases the likelihood that salient oscillatory patterns are well captured.

Decomposition alone, however, does not explain the result. With the same multi-basis front-end, CPE reaches only 51.80% and requires 49.30 min. The contrast shows that the back-end entropy must also match the representation. DE is well suited to multi-basis sub-band analysis because concentric decomposition generates many sub-band sequences, making a low-cost entropy core essential; moreover, DE remains sensitive to structural changes in oscillatory patterns without relying solely on ordinal rankings.

The success of CDE therefore reflects the coupling between multi-basis front-end decomposition and a compatible, low-cost entropy core, rather than the decomposition stage alone.

#### 4.4.3. Time-Frequency Transformations

The two-dimensional hierarchical time–frequency methods further show that domain transformation can be beneficial when the entropy measure matches the transformed representation. MHTF-FE2D reaches 76.27%, whereas MHTF-PE2D reaches only 50.93%.

This contrast suggests that fuzzy similarity is better suited than ordinal ranking for comparing complex 2D time–frequency patterns. In transformed domains, the entropy core must match the geometry and continuity of the resulting representation.

Taken together, HMPE, CDE, and MGGDIPE point to a common design principle: strong methods enrich the signal representation before entropy evaluation. Hierarchical and concentric frameworks preserve information across frequency bands, while adaptive symbolization retains amplitude and distributional information that purely ordinal descriptors suppress. In this benchmark, front-end information enrichment is more effective than post hoc statistical refinement of an information-reduced representation.

### 4.5. Other Representative Variants

This subsection examines methods based on less conventional ideas, including higher-order statistics, fractional calculus, phase analysis, and alternative signal transformations.

#### 4.5.1. Performance of Generalized Multi-Scale Methods

Generalized multi-scale methods replace mean-based coarse-graining with higher-order moments in order to capture volatility and asymmetry.

On their own, however, the basic GME variants perform poorly: GMPE and GMDE achieve only 33.40% and 34.00%, respectively. Higher-order moments are more volatile and therefore more vulnerable to the instability caused by standard coarse-graining.

Performance improves substantially once these methods are combined with composite or refined composite stabilization. GCMFE reaches 72.43%, and GRCMDE reaches 76.07%.

The implication is that higher-order dynamics can be informative, but only when the underlying estimates are stabilized sufficiently.

#### 4.5.2. Information Loss from Radical Transformations (MPEG and RCZMWFE)

Some mathematically elaborate transformations show the opposite pattern, combining high computational cost with limited diagnostic benefit. MPEG achieves only 37.77% while requiring 53.70 min, and RCZMWFE reaches 46.40% with an extreme runtime of 542.59 min. This should not be interpreted simply as an implementation failure. Rather, it may indicate a mismatch between the transformed representation and the physical characteristics of gear fault vibration signals. MPEG emphasizes graph topology over precise temporal and amplitude structure, whereas RCZMWFE uses zoom/minimum-based operations that may suppress high-amplitude fault impulses.

#### 4.5.3. Front-End Intelligence: MGGDIPE

MGGDIPE provides the opposite example. It reaches 73.30% with a runtime of only 1.67 min, outperforming several more elaborate methods. Its success supports a front-end intelligence strategy: adaptive generalized-Gaussian normalization and improved symbolization create a richer input representation before entropy is evaluated [55]. This result again supports an enrich-then-analyze strategy rather than repeated post hoc refinement of a less informative representation.

#### 4.5.4. Fractional Methods

Fractional methods show mixed behavior. MFDE improves on MDE by 3.97%, whereas MFFE is 9.93% lower than MFE. The fractional parameter can change sensitivity to long-range dependence, but its benefit is not uniformly robust across entropy types. This again reflects compatibility: changing the operator order is useful only when the altered sensitivity matches the structure of the encoded signal.

#### 4.5.5. Phase Methods

Phase-domain methods are similarly heterogeneous. FMPPE and FMPDE perform poorly, whereas FMPFE reaches 69.63%.

This pattern suggests that the phase signal is not intrinsically uninformative; rather, its usefulness depends on the entropy core. In this dataset, FE captures diagnostically relevant phase regularity more effectively than PE or DE, likely because its distance-based similarity metric is better aligned with the continuous temporal structure of phase dynamics.

#### 4.5.6. Transition Methods

Transition entropy methods also illustrate back-end dependence. CMTPE and CMTDE perform well, reaching 68.47% and 74.13%, whereas CMTFE remains low at 42.60%. Modeling state-to-state evolution can be informative, but its benefit depends on whether the chosen entropy core can exploit transition probabilities without disrupting its native similarity or pattern-statistic mechanism.

### 4.6. Practical Summary

The benchmark results can be distilled into several practical principles for multi-scale method selection and development.

Four principles emerge:

(1) Prioritize statistical robustness. The progression from MSE to CME to RCME shows that stable estimation across shifted or refined paths can improve reliability, but small accuracy differences should still be interpreted together with standard deviations and, where possible, formal non-parametric tests.
(2) Preserve information across the spectrum. The strong results of hierarchical and concentric methods indicate that diagnostically useful features are widely distributed and should not be suppressed by low-pass coarse-graining.
(3) Match enhancements to the base entropy. In this benchmark, variance-based weighting consistently benefits PE, but it degrades FE and does not reliably improve DE.
(4) Treat efficiency as a primary criterion rather than a secondary one. High runtime does not guarantee high accuracy; CDE is both highly accurate and fast, whereas MPEG and RCZMWFE are costly and ineffective.

Representative methods are grouped by accuracy and computational cost in Table 3.

For engineering use, method selection should depend on deployment constraints. High-accuracy but costly methods are better suited to offline diagnosis or post-event analysis, whereas low-cost and stable methods are preferable for online monitoring, embedded systems, and edge devices. Fault type and fault severity should also be considered as a third practical dimension: a method that works well for one fault mechanism or degradation stage may not be optimal for another. Practical method-selection guidance for four deployment scenarios is provided in Table 4.

## 5. Future Directions

The benchmark clarifies the performance patterns observed under the controlled benchmark of multi-scale entropy methods and indicates where future development is most likely to be productive.

### 5.1. Integration with Deep Learning

Multi-scale methods are increasingly used together with deep learning classifiers, but the two paradigms often remain only sequentially connected, which can limit their potential synergy. Both paradigms also have their own limitations. This study therefore advocates a deeper integration of multi-scale analysis with deep learning, moving beyond simple sequential use toward unified frameworks that combine data-driven representation learning with the physical and theoretical constraints offered by multi-scale analysis [61,62].

Three integration paths appear particularly promising:

(1) Incorporating multi-scale entropy (such as the high-performing CDE or HMPE from this benchmark) as components of the loss function or regularization terms to constrain the neural network learning process. This approach aids in controlling model complexity, enhancing generalization capabilities, and guiding the model toward learning more informative representations.
(2) Employing deep networks (e.g., autoencoders) to learn compressed, information-dense representations from raw signals, which then serve as efficient inputs for multi-scale entropy analysis of the system’s underlying dynamics.
(3) Embedding hierarchical decomposition or adaptive filtering modules directly into end-to-end architectures to retain physical interpretability while improving representation learning.

These directions warrant systematic validation, but the benchmark suggests that front-end structure and entropy compatibility will remain important even in deep learning settings.

A practical AI integration workflow can be summarized as follows: vibration acquisition → signal segmentation → multi-scale entropy feature layer → shallow or deep diagnostic model → uncertainty/health-state output → edge or cloud deployment. In this workflow, entropy features may serve as interpretable inputs to conventional classifiers, auxiliary channels for deep networks, self-supervised pretext features, or lightweight health indicators for digital twin and edge-monitoring systems.

This integration should be validated carefully rather than assumed. Deep learning, foundation models, and physics-informed models may improve representation learning, but entropy features remain valuable when interpretability, low-cost deployment, and physically meaningful monitoring indicators are required.

### 5.2. High-Return and Low-Return Directions

Future methodological development should be selective: effort should concentrate on designs that enrich the signal representation before entropy calculation and should move away from transformations with weak physical justification.

#### 5.2.1. High-Return Directions

Representative strong performers such as CDE, HMPE, and MGGDIPE share a common principle: front-end information enrichment is more effective than back-end statistical repair.

This observation points to two high-return directions:

(1) Adaptive signal structuring. Rather than relying on fixed filter banks, future work could learn task-specific wavelet bases or decomposition filters directly from data.
(2) Richer symbolization. Future entropy designs should encode more than ordinal order, for example local amplitude, phase, instantaneous frequency, or volatility, so that the symbolic representation retains more of the underlying dynamics. Hybrid preprocessing pipelines may also remain useful when they improve, rather than obscure, the diagnostic structure of the signal [55,63,64].

#### 5.2.2. Low-Return Directions

By contrast, increasingly elaborate transformations appear to offer limited return when they do not preserve the physical meaning of the vibration signal. The weak results of MPEG and RCZMWFE indicate that mathematical novelty alone is insufficient; successful transformations should be motivated by explicit hypotheses about which fault-relevant signal properties are preserved.

### 5.3. Robustness, Dataset Dependence, and Edge Deployment

Several limitations should be considered when interpreting the benchmark. First, all experiments use the XJTU-Gearbox dataset and a single signal channel; therefore, the observed ranking may change on CWRU, Paderborn, MFPT, or other datasets with different sensors, sampling rates, loads, speeds, and fault severities. Second, the fixed KNN classifier isolates entropy feature quality but does not prove that the same ranking will hold for SVM, Random Forest, XGBoost, LightGBM, or deep classifiers. Third, practical diagnosis also requires robustness to noise, missing samples, operating condition variation, and parameter perturbation. These factors should be evaluated in future cross-dataset and cross-condition studies.

For edge-computing applications in gear and bearing diagnosis, the accuracy–cost trade-off is especially important. Methods intended for embedded monitoring should have stable accuracy, low runtime, limited memory demand, and minimal hyperparameter tuning. This requirement favors entropy designs that enrich diagnostic information before entropy calculation while avoiding transformations that are computationally expensive but weakly linked to physical fault mechanisms.

## 6. Conclusions

The following conclusions are drawn within the controlled benchmark setting of the XJTU-Gearbox dataset, a fixed KNN classifier, and the parameter choices described in the experimental protocol.

This study benchmarked 85 multi-scale entropy methods on the XJTU-Gearbox dataset and identified the main design principles governing diagnostic accuracy and computational efficiency.

(1) Hierarchical methods often outperform conventional coarse-graining because they preserve both low- and high-frequency components rather than discarding high-frequency fault information.
(2) Refined composite strategies yield more stable estimates than original or composite multi-scale methods because they aggregate intermediate statistics before applying the nonlinear entropy operator.
(3) Enhancement mechanisms must be compatible with the base entropy: variance-based weighting benefits PE in this benchmark, but degrades FE and does not consistently improve DE.
(4) Computational cost is not a reliable proxy for diagnostic value; some of the most expensive methods perform poorly, whereas CDE is both fast and accurate.
(5) Representative strong performers such as CDE, HMPE, and MGGDIPE show that improving the signal representation before entropy evaluation is more productive than increasingly elaborate back-end refinement.

These findings should not be interpreted as a universal ranking across all datasets, classifiers, operating conditions, or fault severities. Future work should extend the benchmark to additional public datasets, complete paired statistical testing when run-level records are available, evaluate robustness under noise and missing data, and examine classifier-independent trends under both shallow and deep diagnostic models.

## Figures and Tables

**Table 1:** Parameter settings of entropy methods and classifier.

Component	Scope	Parameters Used in the Benchmark	Fairness Rationale
Data segmentation	All methods	Segment length = 2048; samples per class = 100; Y-direction vibration signal	All methods receive the same input samples.
Multi-scale setting	Main benchmark	Scales = 20 unless a method-specific script defines another scale range	A common scale length is used while preserving method-specific definitions.
Data split and repetition	All methods	70/30 train/test split; 20 repeated runs	Mean and standard deviation are reported under the same protocol.
Classifier	All entropy features	KNN; k = 5; Euclidean distance; no feature selection; no distance weighting	Classifier effects are kept fixed so feature quality is emphasized.
PE-family methods	PE, MPE, CMPE, RCMPE, GCMPE, etc.	Typical main settings: embedding dimension m = 6; delay tau = 1; scales = 20	PE parameters follow permutation-pattern construction practice.
FE-family methods	FE, MFE, CMFE, RCMFE, GCMFE, etc.	Typical main settings: m = 2; r = 0.15; fuzzy power *n* = 2; tau = 1; scales = 20	Tolerance and fuzzy power are FE-specific and have no direct PE/DE counterpart.
DE-family methods	DE, MDE, CMDE, RCMDE, GCMDE, etc.	Typical main settings: m = 3; tau = 1; sigma = 30; scales = 20	The mapping/class parameter sigma is DE-specific and should not be numerically unified with PE/FE parameters.
Special variants	Fractional, weighted, wavelet, graph, interpolation, hierarchical, and 2D methods	Method-specific alpha, wavelet basis, hierarchy level, interpolation rule, or variant-specific m/scales retained	Recommended parameters avoid unfairly weakening or over-tuning any family.

**Table 2:** Comprehensive benchmark results of multi-scale entropy implementations grouped by framework and entropy core.

Method Framework	PE	FE	DE
Multi-scale	[23] 53.20 ± 2.80/6.52	[22] 62.63 ± 2.83/2.73	[21] 67.70 ± 3.34/0.18
Composite Multi-scale	[25] 63.13 ± 3.22/41.22	[25] 72.77 ± 2.26/42.68	[25] 78.33 ± 2.61/0.94
Modified Multi-scale	[24] 58.93 ± 2.22/12.46	[24] 65.87 ± 3.64/77.68	[24] 67.10 ± 3.25/0.42
Multi-scale Weighted	[27] 56.77 ± 3.60/5.26	[27] 44.03 ± 3.04/3.74	[21] 60.57 ± 2.57/0.32
Composite Multi-scale Weighted	[28] 68.63 ± 2.16/23.32	[28] 69.20 ± 2.84/9.89	[28] 68.10 ± 2.90/2.10
Refined Composite Multi-scale	[29] 70.43 ± 1.86/37.50	[29] 73.70 ± 2.90/38.84	[29] 79.47 ± 2.52/1.12
Refined Composite Multi-scale Weighted	[30] 73.17 ± 2.95/36.04	[30] 58.53 ± 3.18/11.72	[30] 65.27 ± 2.20/3.12
Refined Composite Downsampling	[32] 68.90 ± 3.32/5.37	[32] 62.63 ± 2.83/3.36	[32] 63.93 ± 3.49/0.38
Refined Composite Global Multi-scale	—	[31] 74.80 ± 2.39/41.97	—
Refined Composite Zoom Multi-scale Weighted	[58] 68.97 ± 3.77/246	[58] 46.40 ± 3.35/542.59	[58] 63.43 ± 2.87/72.53
Generalized Multi-scale	[36] 33.40 ± 2.56/8.84	[36] 46.10 ± 3.92/5.61	[36] 34.00 ± 4.37/0.18
Generalized Composite Multi-scale	[34] 43.90 ± 3.01/12.25	[34] 72.43 ± 2.72/39.81	[34] 40.30 ± 3.39/1.06
Generalized Refined Composite Multi-scale	[35] 63.13 ± 3.22/15.42	[35] 69.93 ± 2.85/12.39	[35] 76.07 ± 2.66/2.74
Multi-scale Generalized Gaussian Distribution Improved	[55] 73.30 ± 3.09/1.67	—	[55] 70.23 ± 3.39/1.82
Hierarchical	[18] 54.13 ± 3.27/0.23	[18] 63.10 ± 3.28/7.27	[18] 54.37 ± 3.16/1.58
Hierarchical Multi-scale	[41] 81.60 ± 2.75/48.43	[41] 70.47 ± 4.05/45.78	[41] 74.07 ± 3.10/1.95
Modified Hierarchical	[40] 58.00 ± 3.12/0.23	[40] 56.07 ± 2.52/245.78	[40] 51.63 ± 3.52/0.26
Modified Hierarchical Amplitude-Aware	[43] 55.63 ± 3.54/10.22	—	—
Two-dimensional Multi-scale Hierarchical Time–Frequency	[48] 50.93 ± 2.16/5.41	[48] 76.27 ± 2.47/39.54	—
Multi-scale Fractional	[17] 60.43 ± 2.84/0.19	[17] 52.70 ± 2.84/3.06	[17] 71.67 ± 2.24/0.14
Refined Composite Multi-scale Fractional	[37] 62.97 ± 3.16/13.61	[37] 73.90 ± 2.74/39.51	[37] 75.80 ± 2.97/0.92
Multi-scale Weighted Fractional	[17] 43.16 ± 2.18/11.02	[17] 43.13 ± 2.75/7.45	[17] 62.93 ± 3.03/0.43
Fractional Multi-scale Phase	[39] 38.30 ± 3.63/1.78	[39] 69.63 ± 3.05/28.80	[39] 37.00 ± 3.58/1.56
Multi-scale Inherent	[50] 58.93 ± 2.22/16.53	[50] 67.40 ± 3.71/195.03	[50] 73.53 ± 2.78/1.12
Composite Interpolation Multi-scale	[51] 55.83 ± 2.97/16.59	[51] 61.33 ± 2.93/33.00	[51] 74.77 ± 2.17/0.45
Composite Multi-scale Transition	[52] 68.47 ± 2.50/4.76	[52] 42.60 ± 3.51/0.63	[52] 74.13 ± 3.41/1.04
Derivative Multi-scale	[53] 53.80 ± 3.92/0.95	[53] 42.20 ± 2.27/27.74	[53] 56.47 ± 2.42/0.52
Graph Multi-scale	[54] 37.77 ± 2.27/53.70	—	—
Fine-to-Coarse Multi-scale	[56] 41.80 ± 3.22/14.11	[56] 49.80 ± 3.17/67.23	[56] 41.53 ± 3.15/13.29
Adaptive Multi-scale Weighted	[57] 61.17 ± 3.98/0.97	[57] 52.00 ± 3.32/4.16	[57] 62.70 ± 3.49/0.65
Concentric	[59] 51.80 ± 3.66/49.30	[59] 73.53 ± 3.08/23.29	[59] 83.47 ± 1.88/0.62

Note: Entries are formatted as [Ref.] Acc. ± SD/Time, where Acc. is the mean classification accuracy (%), SD is the standard deviation over repeated trials, and Time is the feature extraction runtime (min). References identify the literature basis of each method family or framework; all metrics were obtained in this benchmark. “—“ denotes that the corresponding entropy-core implementation was not included.

**Table 3:** Representative accuracy–cost quadrant analysis of multi-scale entropy methods.

	Low Cost	High Cost
High Accuracy	(1) Concentric Diversity Entropy (2) Hierarchical Multi-scale Diversity Entropy (3) Multi-scale Fractional Diversity Entropy (4) Multi-scale Generalized Gaussian Distribution Improved Diversity Entropy	(1) Hierarchical Multi-scale Permutation Entropy (2) Two-dimensional Multi-scale Hierarchical Time–Frequency Fuzzy Entropy (3) Refined Composite Multi-scale Global Fuzzy Entropy (4) Refined Composite Multi-scale Weighted Permutation Entropy
Low Accuracy	(1) Multi-scale Diversity Entropy (2) Multi-scale Permutation Entropy (3) Hierarchical Permutation Entropy (4) Multi-scale Weighted Permutation Entropy	(1) Refined Composite Zoom Multi-scale Weighted Fuzzy Entropy (2) Graph Multi-scale Permutation Entropy (3) Modified Hierarchical Fuzzy Entropy (4) Composite Multi-scale Permutation Entropy

Note: High accuracy denotes mean accuracy ≥ 62.93%, and low cost denotes runtime ≤ 5.61 min; both thresholds are the medians across the 85 methods in Table 2.

**Table 4:** Practical method-selection guidance for entropy method deployment.

Scenario	Preferred Method Property	Practical Implication
Offline diagnosis	Higher accuracy and richer representation	More complex methods are acceptable if computation is not time-critical.
Online monitoring	Low runtime and stable repeated-run behavior	Fast methods such as efficient DE-based or hierarchical variants are preferable.
Embedded/edge deployment	Low feature extraction cost and limited hyperparameter tuning	Simple fixed protocols reduce memory and computation burden.
Uncertain fault severity	Robustness across classes and scales	Future work should report fault-type- and severity-aware trade-offs.

## Data Availability

Data will be made available on request.
